# A case of human infection by H3N8 influenza virus

**DOI:** 10.1080/22221751.2022.2117097

**Published:** 2022-09-28

**Authors:** Xin Tan, XiaoTian Yan, Yang Liu, Ying Wu, Ji Yang Liu, Mi Mu, Jie Zhao, XiangYun Wang, Jie Qiong Li, Long Wen, Peng Guo, Zhi Guo Zhou, Xiu Bin Li, Peng Tao Bao

**Affiliations:** a Department of pediatric, The First Hospital of Changsha, Changsha, People’s Republic of China; b Department of Paediatrics, Zhumadian Central Hospital, Zhumadian, People’s Republic of China; c Department of Clinical laboratory, The First Hospital of Changsha China, Changsha, People’s Republic of China; d Changsha Municipal Health Commission, Changsha, People’s Republic of China; e Pulmonary and Critical Care Medicine Faculty, The Eighth Medical Center of Chinese PLA General Hospital, Beijing, People’s Republic of China; f Department of Psychiatry Ward 5th, Zhumadian Second People's Hospital, Zhumadian, People’s Republic of China; g Department of Urology, The Third Medical Center of Chinese PLA General Hospital, Beijing, People’s Republic of China

Dear Editor,

Avian influenza virus (AIVs) is an influenza A virus belonging to the family *Orthomyxoviridae* which consists of eight single-stranded negative-sense RNA fragments.

AIVs can infect a broad range of hosts, including humans and birds, and this feature has caused increasing concern worldwide due to the ability of the viruses to cross the species barrier and cause severe disease in humans [1–3]. AIVs are subtyped according to the antigenicity of the surface glycoproteins. These subtypes include 16 hemagglutinin (HA) subtypes (H1–16 in wild birds) and 9 neuraminidase (NA) subtypes (N1–9 in wild birds) [4, 5]. Current epidemiological data suggest that the H3 is the most common mammalian-adapted HA subtype of influenza A, thereby infecting humans, pigs, dogs, horses, cats, seals, and donkeys [6]. Among the H3 subtypes, H3N8 is one of the most commonly found subtypes in wild birds, and has sparked particular interest in research due to its ability for cross-species transmission in mammalian hosts such as horses and dogs, and even in swine under experimental conditions. Although the transmission of H3N8 viruses to mammals has been widely reported, the first case of human infection with the H3N8 virus was only recently reported [7]. Here, we report a human case of infection with a novel, reassorted avian influenza A virus, H3N8.

The patient was a 5-year-old child with no significant medical history who developed fever and cough on 9 May 2022. On May 10, he was admitted to the hospital into the fever department of the First Hospital of Changsha, presenting with a fever of 40°C, chills, sore throat, fatigue, and a runny nose**.** A swab was collected on the same day of admission, and a novel coronavirus nucleic acid test returned a negative result. After two days of self-medication, his condition and respiratory symptoms improved significantly. However, on May 13, the swab that was collected on May 10 was found to be positive for influenza A N8 by real-time PCR (RT–PCR) through routine influenza surveillance. On May 15, the RT–PCR re-examination tested positive for influenza A N8 again, and the influenza A (H3N8) full genome sequence was obtained from the initial swab collected on May 10.

On May 17 (9 days after disease onset), the patient was hospitalized to undergo isolated treatment in the same hospital, whereby oseltamivir and supportive treatment were administered (Figure 1A, Supplemental Table 1). On admission, a physical examination revealed a normal body temperature of 36.8°C. His blood pressure was 100/70 mmHg, pulse rate was 94/min, and air oxygen saturation level was 99%. A swab was once again collected and RT–PCR re-examination returned a positive result for influenza A N8, but was negative for 14 other common respiratory pathogens as described by Li et al. [8]. No influenza A N8 RNA was found in the patient’s father’s swab collected on the same day, despite his close contact with his son. The patient’s father had no clinical manifestations, and he was only further tested for the novel coronavirus nucleic acid, with negative results returned. A computed tomography (CT) scan of the patient’s chest conducted on the same day did not show pneumonitis or other lung pathology (Supplemental Figure 1).

**Figure 1.**
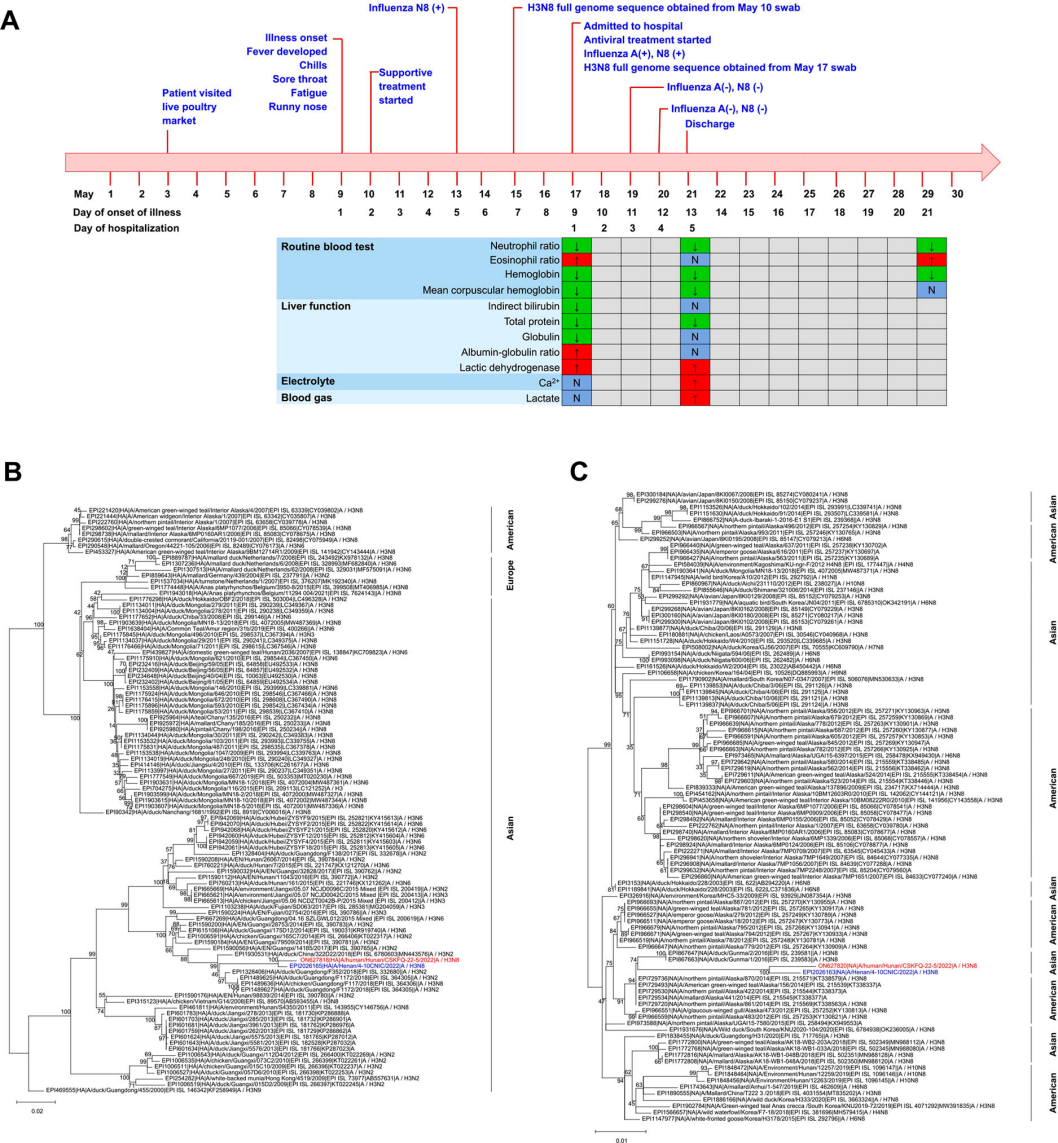
A 5-year-old boy infected with Avian influenza A (H3N8) virus. (A)Timeline of the clinical course of the patient, identification of causative pathogen and laboratory indicators during hospitalization. ↓ =  below normal range. N = normal range. + =  positive. - = negative. Phylogenetic trees for the full-length HA (B) and NA (C) genes of H3 and N8 subtype influenza viruses. A/Hunan/CSKFQ-22-5/2022(H3N8) virus was indicated with red colour and A/Henan/4-10CNIC/2022(H3N8) was indicated with blue colour.

By PCR-based sequencing conducted on the swab collected on May 17 using a pair of universal full-length primers (MBTuni-12 and MBTuni-13), we obtained all the eight segments of the influenza A/H3N8 virus (A/Hunan/CSKFQ-22-5/2022(H3N8)); GenBank accession number (ON627815-ON627822) [9]. Phylogenetic analyses of the HA gene showed high homology (98.62%) to the previously reported human H3N8 virus A/H3N8/Henan/4-10CNIC/2022 (Figure 1B and C). The most closely related virus to the NA gene was also to A/H3N8/Henan/4-10CNIC/2022 (with a nucleotide identity of 98.59%), together with H3N8 viruses in various species of birds in North America in 2014 and Japan in 2016 (Supplemental Figure 2) [10].

However, other internal genes showed high homology to those belonging to the chicken-origin H9N2 subtype (Supplemental Figure 2). This might indicate that CSKFQ-22-5 was distinct from the previously reported A/H3N8/Henan/4-10CNIC/2022, and that it had undergone complex reassortment events.

Epidemiological investigation found that the patient had visited a live poultry market (Fucheng fresh market, Kaifuqu, Changsha,) with his grandmother on May 3, before the onset of his illness, though there was no direct contact with live poultry during this period. The visit to this live poultry market was the only means of contact with poultry before the onset of the patient's illness, and there were no pets or poultry kept at his home. Samples taken from his close contacts (his father, mother, grandmother, and grandfather) all tested negative for the N8 nucleic acid. Specific detection of N8 RNA by RT–PCR revealed negative results for all of the 23 samples collected from chickens and the surfaces contained within the exposure site, as well as two other markets near the exposure site (the Chenjiadu farm produce market and Hehuachi fresh market) (Supplemental Figure 3). The samples collected were specifically obtained from oral swabs of the chickens, chicken feces, chicken cages, chicken drinking water, surfaces of the work benches where chickens were handled, and the environment around the stalls.

H3N8 is the most common subtype of avian influenza, and the known hosts for H3N8 IAVs (type A influenza viruses) include wild birds, poultry, horses, cats, dogs, and seals. Like most of the zoonotic pathogens, H3N8 is not well adapted to humans and only emerge sporadically through spillover events. To date, there is only one report on human infection with H3N8 since its first identification in Florida in 1963 [11]. The first H3N8 patient, a child with acute respiratory distress syndrome, was identified in Henan province of China in April 2022 [7]. The patient presented with fever and lethargy, and then quickly went on to develop severe acute respiratory distress syndrome [7]. Although the overall outcome of the patient was not clear, chest CT showed extensive interstitial changes and consolidation in the lungs, with small airway involvement on the 11th day of admission. The H3N8 can still be detected in the lavage fluid on the day 14 of his illness, indicating that the clearane time of H3N8 in the patient was prolonged. For the currently described case, however, the boy featured the initial symptoms of influenza-like-illness, but this was then followed by rapid recovery and long-lasting laboratory abnormalities 21 days after the disease onset (Supplemental Table 2). This suggests that, even if such a patient could be cured and discharged, it would still be necessary to follow up on the patient's laboratory abnormalities for an extended period. This knowledge might be beneficial for the effective treatment and control of a future H3N8 outbreak.

At present, there is a large amount of research existing on the adaptation of AIVs to mammals. In recent years, studies have shown that the receptor binding site changes Q226L and G228S can increase the binding of AIVs to the human sialic acid α-2,6 galactose receptor [12]. Two amino acid changes in the polymerase PB2 protein, E627K and D701N, are determinants for host range and virulence [13]. In addition, it has been reported that the N30D and T215A mutations in the *M* gene and the P42S mutation in the *NS* gene can increase virulence in animal models [14]. In our study, the N30D and T215A mutations in the *M* gene and the P42S mutation in the *NS* gene, which was also found in A/H3N8/Henan/4-10CNIC/2022, were all detected in A/H3N8/Hunan/CSKFQ-22-5/2022 (Supplemental Table 3). However, different PB2 residue 627 was observed between A/H3N8/Henan/4-10CNIC/2022 and A/H3N8/Hunan/CSKFQ-22-5/2022. So far, there are no reports on the impact of a single residue substitution in PB2 residue 627 of H3N8 on host defense, so further studies were warranted in future.

In reviewing the results of this study, one must also keep several potential limits in mind. First, it has been reported that genetic variations within the host’s genomes play a crucial role in determining the course and severity of zoonotic IAV infections in humans [15]. Future studies are warranted to understand the roles of these human genetic variations in the infectability and disease severity of H3N8. Second, despite of the attempts in the *in vitro* cultivation, no isolation of H3N8 was achieved. The potential virological characteristics underlying the human infection of H3N8 warrants further confirmation in future studies.

In summary, we report the first known human infection with H3N8 in the Hunan Province, China. This was a case of human infection with the H3N8 avian influenza virus. Although this particular case of infection can be considered sporadic and without human-to-human transmission, we stress the importance of paying increased attention to zoonotic H3N8 viruses such as this one.

## Supplementary Material

Supplemental Material
