# Supplementary material for: A case of human infection by H3N8 influenza virus
Source: Emerg Microbes Infect. 2022 Sep 28;11(1):2214–7. doi: 10.1080/22221751.2022.2117097 (PMC9542523; doi:10.1080/22221751.2022.2117097)
Supplement: Supplemental Material [file TEMI_A_2117097_SM8316.docx]

**Supplemental Figure 1. The patient’s Chest CT scan on May 17, 2022.**


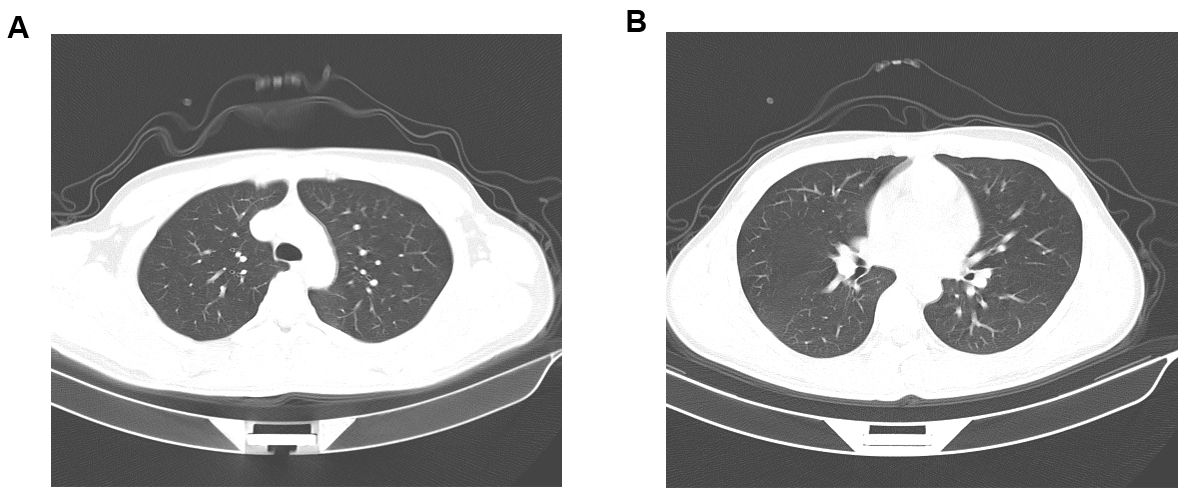


**Supplemental Figure 2. Phylogenetic trees of PB2 (A), PB1 (B), PA (C), NP (D), M (E) and NS (F) genes of the novel avian influenza A (H3N8) virus.** A/Hunan/CSKFQ-22-5/2022(H3N8) virus was indicated with a red color and A/Henan/4-10CNIC/2022(H3N8) was indicated with a blue color.

**
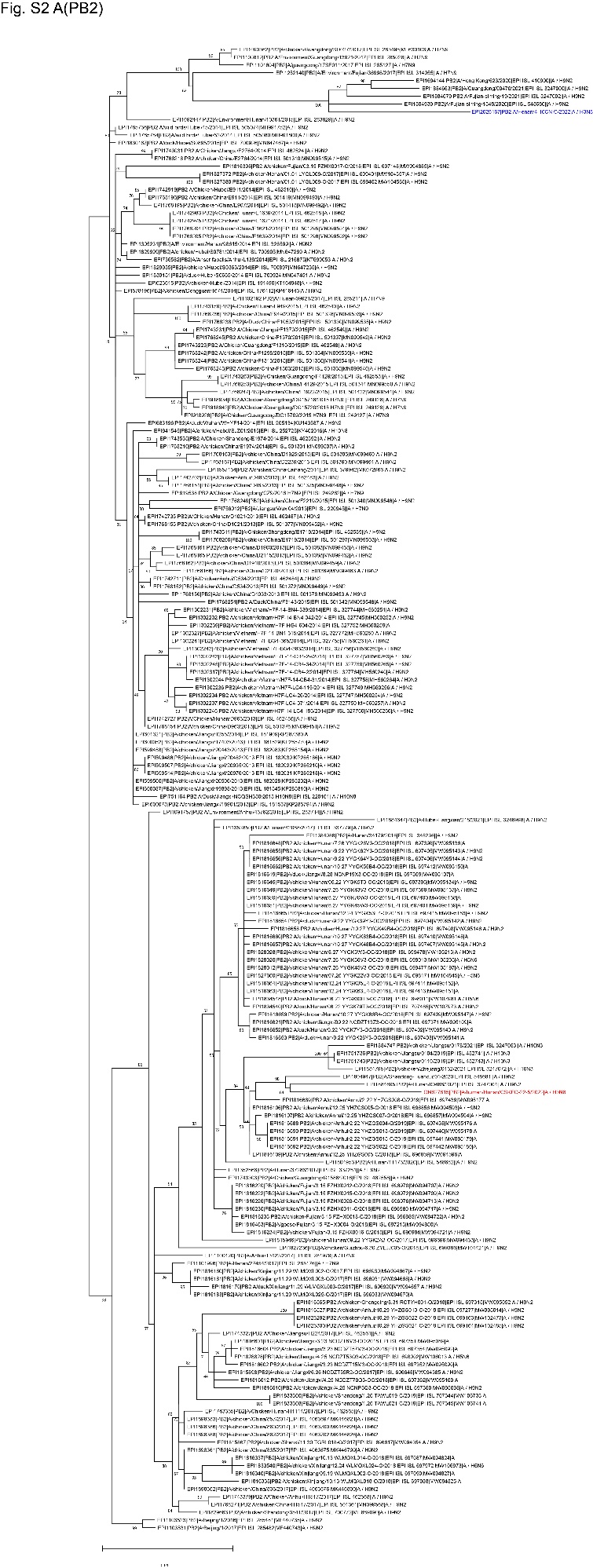
**

**
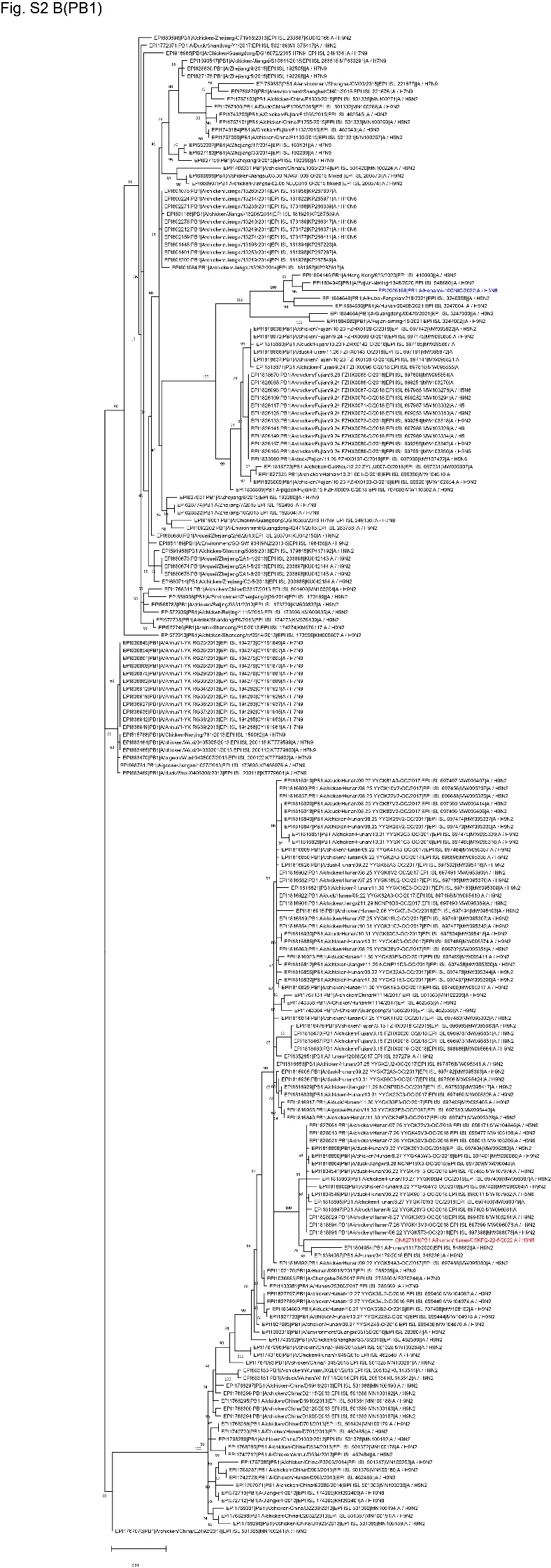
**

**
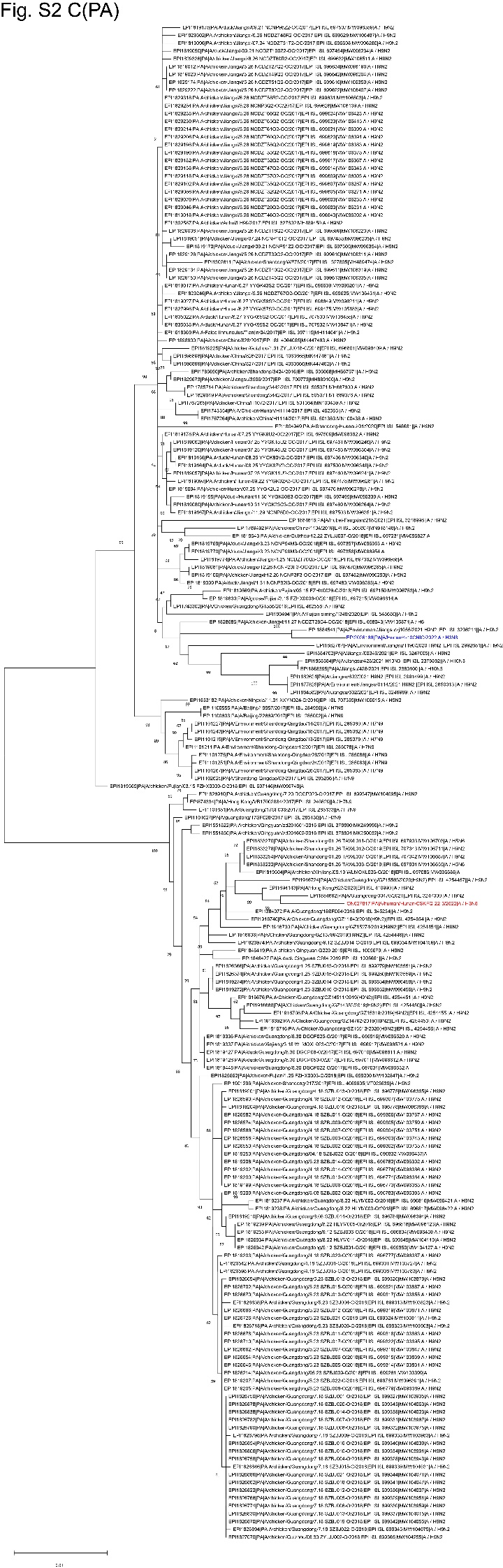
**

**
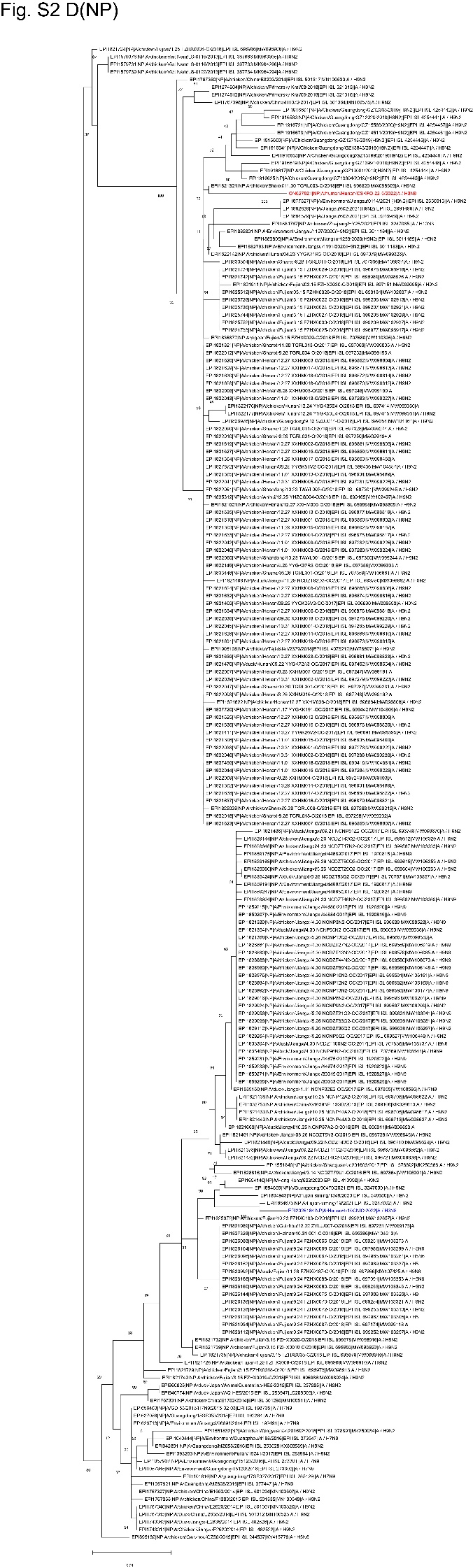
**

**
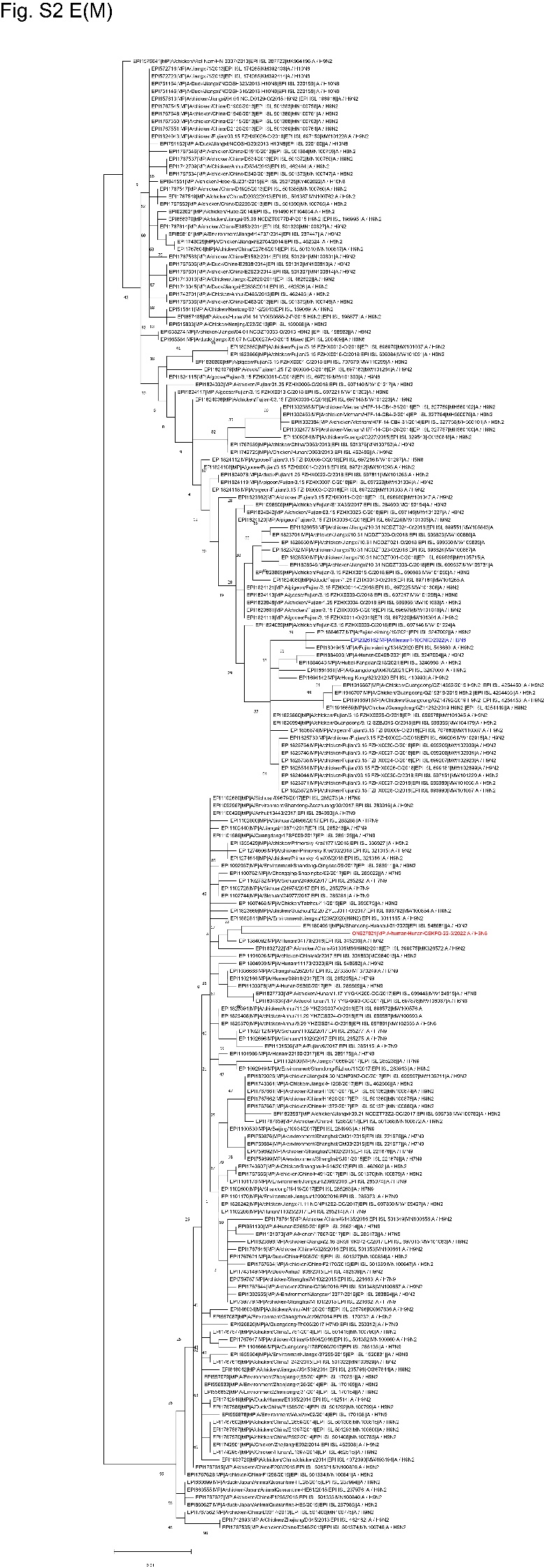

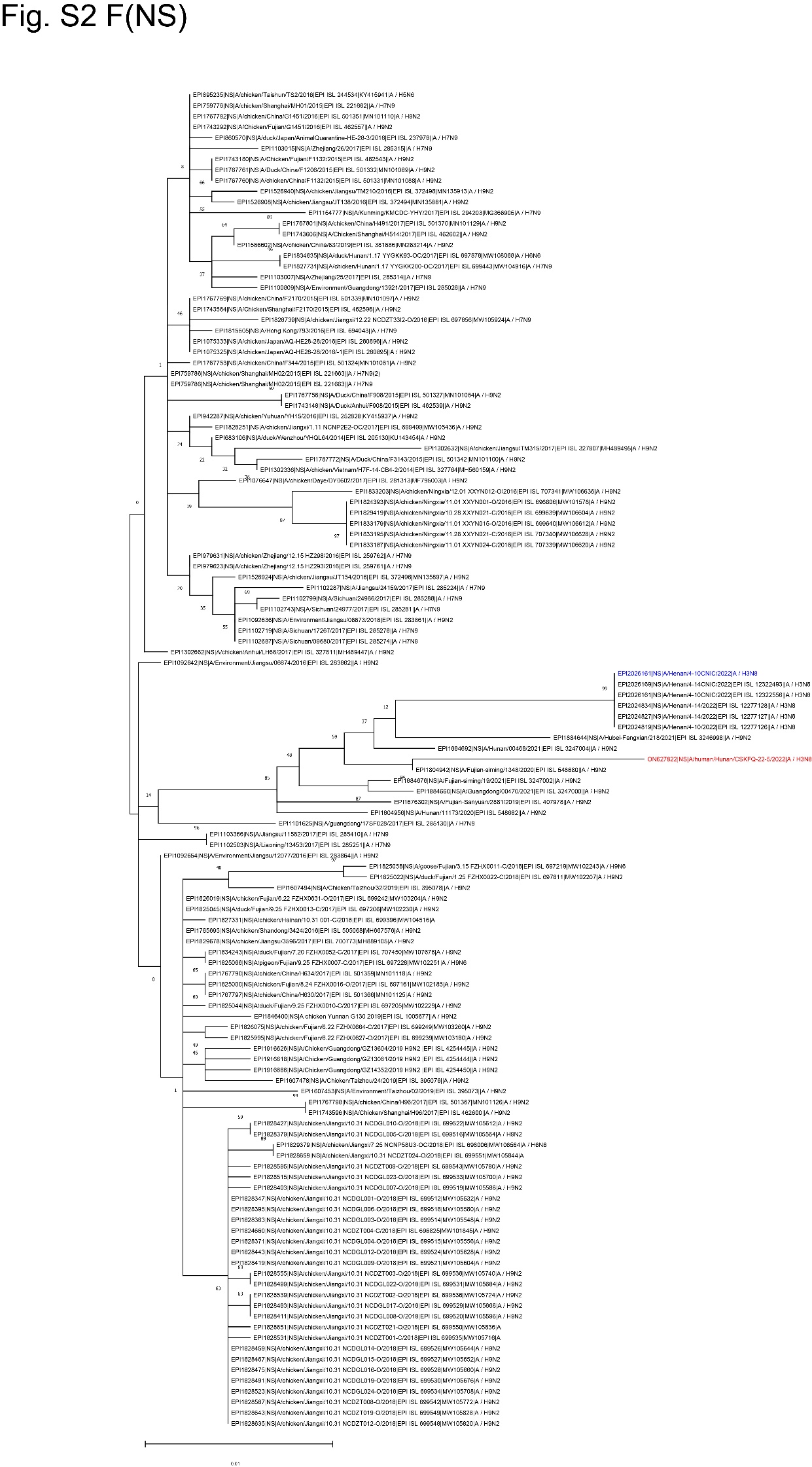
**

**Supplemental Figure 3. The H3N8-infected patient’s house, hospital and surrounding environment, Kaifuqu, Changsha, Hunan Province, China. A.** The location of the patient is in Kaifuqu, Changsha, Hunan Province, China; **B.** Satellite image of the house of the patient (marked in red square), the hospital (marked in red triangle) where the swab was collected at May 10 and was found to be influenza A N8 positive on May 13th analysis, the exposure site (marked in orange circle) and the sampling sites (marked in circles with different colors).

**
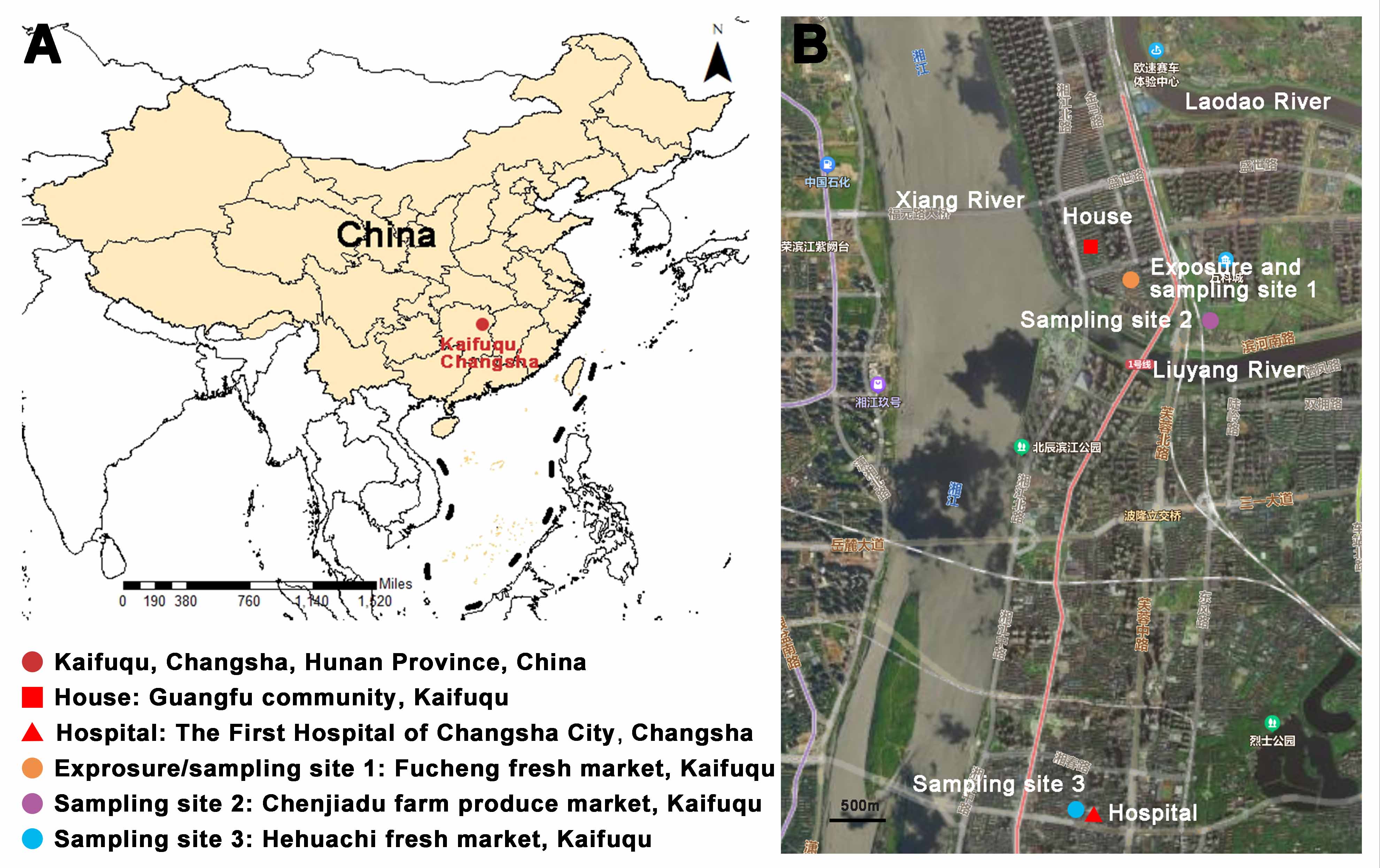
**

**Supplemental Table 1.** **Signs and treatments of the patient.**

| **Signs and treatment** | | |
| --- | --- | --- |
| Fever | | Yes |
|  | Temperature on admission (°C) | 36.8 |
|  | Highest temperature (°C) | 40.0 |
| Antiviral treatment |  |  |
| Oseltamivir phosphate granules | Hospital day 1-5 | 45mg, twice a day |
| Chinese medicine |  |  |
| [Liquorice](javascript:;) [tablet](javascript:;)s | Hospital day 2-5 | 3g (crude drug), twice a day |
| Polygonum cuspidatum | Hospital day 2-5 | 10g (crude drug), twice a day |
| Radix isatidis | Hospital day 2-5 | 5g (crude drug), twice a day |
| Lophatherum gracile | Hospital day 2-5 | 20g (crude drug), twice a day |
| Pulvistalci | Hospital day 2-5 | 9g (crude drug), twice a day |
| Stachyurus pith | Hospital day 2-5 | 10g (crude drug), twice a day |
| [Rhizoma](javascript:;) [Pinellinae](javascript:;) [Praeparata](javascript:;) | Hospital day 2-5 | 3g (crude drug), twice a day |
| Jiang Magnolia Puerariae | Hospital day 2-5 | 6g (crude drug), twice a day |
| Coix Seed | Hospital day 2-5 | 30g (crude drug), twice a day |
| Amomum kravanh | Hospital day 2-5 | 5g (crude drug), twice a day |
| Bitter almond | Hospital day 2-5 | 5g (crude drug), twice a day |

|  | **Normal range** | **May 17**  **(Hospital Day 1)** | **May 22**  **(Hospital day 5)** | **May 29** |
| --- | --- | --- | --- | --- |
| **Routine blood test** |  |  |  |  |
| Neutrophil ratio (%) | 50-70 | 39.7(↓) | 39.4(↓) | 44.2(↓) |
| Eosinophil ratio, EOS% (%) | 0.5-5 | 6(↑) | 5 | 5.2(↑) |
| Hemoglobin, HGB (g/L) | 120-160 | 117(↓) | 118(↓) | 114(↓) |
| Mean corpuscular hemoglobin, MCH (pg) | 27-34 | 26.8(↓) | 26.6(↓) | 27.3 |
| **Liver function** |  |  |  |  |
| Indirect bilirubin (umol/L) | 5.1-13.7 | 1.9(↓) | 4.4 |  |
| Total protein (g/L) | 66-87 | 64.6(↓) | 65.3(↓) |  |
| Globulin (g/L) | 20.2-29.5 | 17.3(↓) | 20.7 |  |
| Albumin-globulin ratio | 1.5-2.5 | 2.73(↑) | 2.15 |  |
| Lactic dehydrogenase (U/L) | 135-225 | 278(↑) | 241(↑) |  |
| **Electrolyte** |  |  |  |  |
| Ca^2+^ (mmol/L) | 2.15-2.5 | 2.45 | 2.51(↑) |  |
| **Blood gas** |  |  |  |  |
| Lactate (mg/L) | 45.05-198.2 | 154.2 | 219(↑) |  |

**Supplemental Table 2. Laboratory test result of the patient**

**Supplemental Table 3. Key molecular characteristics of the A(H3N8) virus identified in this study.**

| Gene | Mutation | A/H3N8/Henan/4-10CNIC/2022 | A/H3N8/Hunan/CSKFQ-22-5/2022 | Mutation comments |
| --- | --- | --- | --- | --- |
| HA | Cleavage site | PEKQTR/GL | PEKQTR/GL | Pathogenic to poultry |
|  | Q226L | Q | Q | RBS position, altered receptor specificity |
|  | G228S | G | G |  |
| PB1 | H99Y | H | H | H5 virus transmissible among ferrets |
| PB2 | Q591K | Q | Q | Enhance replication efficiency and Increase virulence in mice |
|  | E627V | K | V | Unknown |
|  | D701N | D | D | Increase virulence in mice and transmission in mammals |
| NP | N319K | N | N | Enhance replication efficiency |
| M | N30D | D | D | Increase virulence in mice |
|  | T215A | A | A |  |
| NS | P42S | S | S | Increase virulence in mice |
